# Control of Directed Cell Migration In Vivo by Membrane-to-Cortex Attachment

**DOI:** 10.1371/journal.pbio.1000544

**Published:** 2010-11-30

**Authors:** Alba Diz-Muñoz, Michael Krieg, Martin Bergert, Itziar Ibarlucea-Benitez, Daniel J. Muller, Ewa Paluch, Carl-Philipp Heisenberg

**Affiliations:** 1 Max Planck Institute of Molecular Cell Biology and Genetics, Dresden, Germany; 2 International Institute of Molecular and Cell Biology, Warsaw, Poland; 3 BIOTEC, Technische Universität Dresden, Dresden, Germany; 4 Department of Biosystems Science and Engineering, Eidgenössische Technische Hochschule Zürich, Basel, Switzerland; 5 Institute of Science and Technology Austria, Klosterneuburg, Austria; University of Cambridge, United Kingdom

## Abstract

Cell shape and motility are primarily controlled by cellular mechanics. The attachment of the plasma membrane to the underlying actomyosin cortex has been proposed to be important for cellular processes involving membrane deformation. However, little is known about the actual function of membrane-to-cortex attachment (MCA) in cell protrusion formation and migration, in particular in the context of the developing embryo. Here, we use a multidisciplinary approach to study MCA in zebrafish mesoderm and endoderm (mesendoderm) germ layer progenitor cells, which migrate using a combination of different protrusion types, namely, lamellipodia, filopodia, and blebs, during zebrafish gastrulation. By interfering with the activity of molecules linking the cortex to the membrane and measuring resulting changes in MCA by atomic force microscopy, we show that reducing MCA in mesendoderm progenitors increases the proportion of cellular blebs and reduces the directionality of cell migration. We propose that MCA is a key parameter controlling the relative proportions of different cell protrusion types in mesendoderm progenitors, and thus is key in controlling directed migration during gastrulation.

## Abstract 2

Analysis of cell migration in vivo combined with biophysical measurements reveals how membrane-to-cortex attachment fine-tunes the type of protrusions formed by cells and, as a consequence, controls directed migration during zebrafish gastrulation.

## Author Summary

Cell migration, like any event involving shape changes, is a mechanical process controlled by complex biochemical pathways. Here, we examine cell migration in developing embryos with a combination of cell biological tools and atomic force microscopy, so as to investigate how cellular mechanical properties control migration. A fundamental step during migration is the formation of a protrusion at the leading edge of the cell. In three-dimensional environments, and particularly in vivo, cells use different protrusion types: spike-like filopodia and flattened lamellipodia, whose growth is driven by actin polymerization, and spherical blebs, which grow because of intracellular pressure pushing on the membrane. It is important to understand how the formation of different protrusion types is mechanically and molecularly controlled, and how the different protrusions specifically contribute to migration. We have addressed this using the zebrafish embryo as a model system. We show that reducing the strength of the attachment between the plasma membrane and the underlying cortical network of actin filaments, or increasing intracellular pressure, increases the proportion of cellular blebs and reduces the directionality of cell migration. Our work reveals that blebs, lamellipodia, and filopodia are not interchangeable and that the relative proportion of each type of protrusion, under the control of mechanical parameters, determines migration directionality during zebrafish gastrulation.

## Introduction

During development of the vertebrate body, progenitor cells must migrate from the site at which they are specified to the site where they will eventually form the different body parts. Cell migration is the direct result of mechanical forces mediating cell shape changes and cell-substrate translocation [1]. Thus, the study of cellular mechanics is a prerequisite for understanding cell migration [2]–[4]. In recent years, most studies of cell migration have focused on its molecular control [5]. To fully understand migration, the molecules controlling cell migration must be linked to the mechanics underlying this process.

The attachment of the plasma membrane to the cytoskeleton (membrane-to-cortex attachment [MCA]) has been proposed to be an important mechanical parameter involved in cell shape changes, such as protrusion formation [6]. MCA is thought to modulate the protrusive activity of cells by providing resistance to the flow of plasma membrane into the expanding protrusion [7].

Several molecules are involved in the regulation of MCA, including Ezrin/Radixin/Moesin (ERM) proteins and class 1 myosins [8],[9]. Studies in mice, *Drosophila melanogaster*, *Caenorhabditis elegans*, and cultured cells have shown that ERM proteins are critical for cell shape control during mitotic cell rounding, cell polarization, cell migration, and cell-cell adhesion [10]–[14]. Likewise, class 1 myosins in both single-celled eukaryotes and metazoans have been implicated in various morphogenetic processes, ranging from actin polymerization and microvilli formation to cell motility [15]. In zebrafish, ERM proteins, and in particular Ezrin, are essential for tissue morphogenesis during gastrulation ([16] and Figure S1; for methods see Text S1), while the role of zebrafish class 1 myosins has not yet been studied. It remains unclear whether the functions of ERM proteins and class 1 myosins in cell and tissue morphogenesis are the direct consequence of MCA modulation, or are linked to other functions of these proteins [15],[17].

To analyze the role of MCA in cell protrusion formation and migration in vivo, we turned to zebrafish anterior axial mesendoderm progenitor cells (prechordal plate progenitors), which during the course of gastrulation migrate from the germ ring margin, where they are specified, towards the animal pole of the gastrula using a combination of different protrusion types [18],[19]. Several signaling pathways, including PDGF/PI3K and Wnt/PCP signaling, have been suggested to control protrusion formation and migration of prechordal plate progenitors [18],[19]. Recently, we showed that ERM proteins are phosphorylated and thus activated in prechordal plate progenitor cells, and are required for prechordal plate morphogenesis ([16] and Figure S1; for methods see Text S1), alluding to the possibility that ERM proteins modulate prechordal plate cell morphogenesis by regulating MCA.

Here, we show that MCA is a critical mechanical parameter determining the proportion of different protrusion types formed by prechordal plate progenitors, and thereby controlling directed migration during zebrafish gastrulation.

## Results

**Figure 1:**
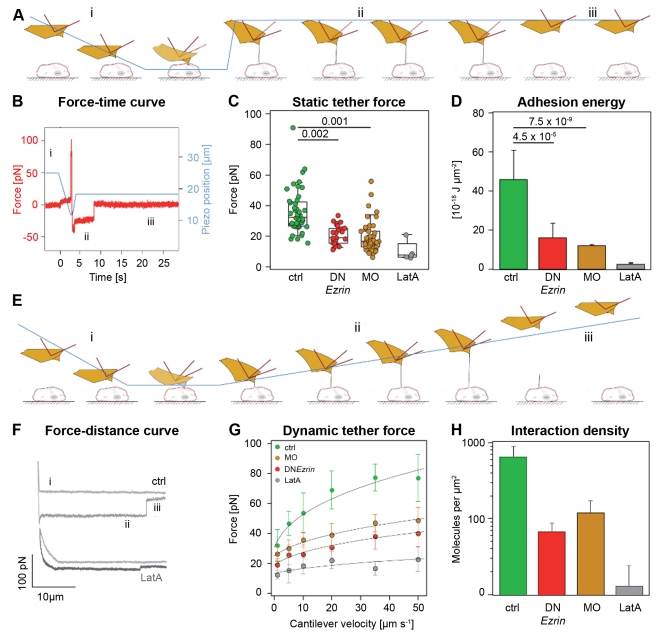
MCA is reduced in ERM-deficient prechordal plate progenitor cells. (A) Schematic outline of the height-clamp experiment. A lectin-coated cantilever is lowered onto a cell. After a short contact time (≈200 ms) the cantilever is retracted 6 µm, and the position is then held constant. Upon retraction, a tether is formed between the tip of the cantilever and the cell membrane. The bending of the cantilever measures the force exerted on the cantilever. i–iii indicate events in the force curve shown in (B): i, cantilever approach and contact formation; ii, tether extrusion; iii, tether rupture. (B) Example force–time curve showing an interaction event. Force is red; piezo position is blue. The vertical step in the force–time trace shows an unbinding event of a tether at 6 µm (clamp set point) above the cell surface. (C) Static tether force in control and ERM-deficient prechordal plate progenitor cells. For numbers of cells probed see Table S1. (D) Median adhesion energy density of control and ERM-deficient prechordal plate progenitor cells determined from *F*_0_ using Equation 1. Error bars indicate absolute deviation of the median. *p*-values were obtained from Mann–Whitney *U* test. (E) Schematic outline of the dynamic tether force spectroscopy experiment. A lectin-coated cantilever is lowered onto a cell. After a short contact time (≈200 ms) the cantilever is retracted 90 µm at different speeds. Upon retraction, a tether can form between the tip of the cantilever and the cell membrane, pulling the cantilever downwards. When a tether unbinds, the cantilever relaxes, causing a vertical force-step in the corresponding force spectrum, as shown in (F). (F) Example force–velocity curves of control and LatA-treated cells acquired by dynamic tether extrusion force spectroscopy. The vertical step in each force curve represents a tether-unbinding event. i–iii refer to events depicted in (E). (G) Median tether forces of control and ERM-deficient prechordal plate progenitor cells as a function of extrusion velocity. For numbers of cells probed see Table S1. (H) Density of membrane-to-cytoskeleton cross-linking molecules of control and ERM-deficient prechordal plate progenitor cells as determined from fits of the tether force–velocity profiles displayed in (G). Error bars indicate error propagated from the fit. For details see Materials and Methods.

To test whether MCA can be modulated in prechordal plate cells by interfering with ERM protein activity, we developed an assay for measuring MCA using atomic force microscopy (AFM), and compared MCA in isolated control and ERM-deficient prechordal plate cells. Control cells were obtained from embryos expressing the Nodal-ligand Cyclops (Cyc), previously shown to induce prechordal plate progenitor cell fate and activate ERM proteins [16],[20]. ERM-deficient cells were obtained from embryos expressing Cyc in combination with either a dominant negative non-phosphorylatable version of *ezrin* (DN*Ezrin* T564A; [21]) or a combination of morpholinos (MOs) targeted against *ezrin* and *moesin-a* to inactivate ERM protein function ([16]; details about MO and controls in Materials and Methods). To quantify MCA, we estimated the adhesion energy density between the plasma membrane and the subjacent cytoskeleton (*W*_0_; Figure 1A and 1B; [22],[23]) by measuring via single cell force spectroscopy [24] the force needed to extrude single lipid-membrane nanotubes (or tethers) from the cell plasma membrane. Various models of tether extrusion have shown that the force required to hold a tether at a constant height (static tether force, *F*_0_; see Figure 1A and 1B) depends on the membrane bending rigidity (κ), the plasma membrane surface tension (σ), and the energy density of MCA (*W*_0_; [22],[23]):
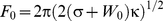
(1)where σ+*W*_0_ is also called apparent surface tension of the membrane (*T*_app_; [22]). By extruding tethers from control and ERM-deficient prechordal plate cells, we found that the static tether force *F*_0_ was significantly reduced in ERM-deficient cells (Figure 1C; Table S1). We then used *F*_0_ to calculate the reduction of apparent tension *T*_app_ in ERM-deficient cells (*T*_app_ = 18 µN•m^−1^, median) compared to control cells (*T*_app_ = 46 µN•m^−1^, median), using a previously determined value for κ, which we assumed was unchanged upon ERM depletion ([25]; details in Materials and Methods). To estimate the corresponding decrease in MCA energy (*W*_0_), we then measured the plasma membrane tension σ by extruding tethers from cells treated with Latrunculin A (LatA) to depolymerize the actin cortex, where *W*_0_ is negligible and thus *T*_app_≅σ [26]. We found *T*_app_ to be strongly reduced in LatA-treated cells (*T*_app_≅2.5 µN•m^−1^≅σ), indicating that σ is small compared to *W*_0_ and contributes very little to *T*_app_ (*W*_0_≅*T*_app_). Using this value of σ, we calculated *W*_0_ and found it to be strongly reduced upon ERM inactivation in prechordal plate cells (Figure 1D).

Inactivating ERM proteins is expected to result in a decrease in the number of molecules cross-linking the cortex to the membrane (cross-linkers). To analyze whether the density of cross-linkers is indeed reduced in ERM-deficient prechordal plate cells, we extruded tethers at varying velocities in control and ERM-deficient cells (Figure 1E and 1F; [22],[27]). The tether pulling force has to counteract the friction of the cross-linkers against lipid bilayer flowing into the tether, and increases with increasing pulling velocities (Figure 1G). A recent model has related pulling force–velocity profiles to the density of cross-linkers and the lipid bilayer viscosity ([23]; details in Materials and Methods). By measuring the diffusion of a palmitoyl-anchored GFP (GAP43-GFP) within the plasma membrane as a reporter of lipid mobility [28], we first verified that the viscosity of the plasma membrane remains unchanged between control and ERM-deficient cells (Figure S2A–S2F; details in Text S1). Using a published value for membrane viscosity (details in Materials and Methods), we then deduced the density of membrane-to-cortex cross-linking molecules from the fits of the force–velocity profiles. We found that control cells displayed about 600 cross-linking molecules per square micrometer, which corresponds to a 41-nm lateral separation between molecules on average (Figure S2G). In ERM-deficient cells, the density of cross-linking molecules was strongly reduced (Figures 1H and S2G), indicating that the reduction of *W*_0_ in ERM-deficient cells is caused by a decrease in the density of active cross-linking molecules.

**Figure 2:**
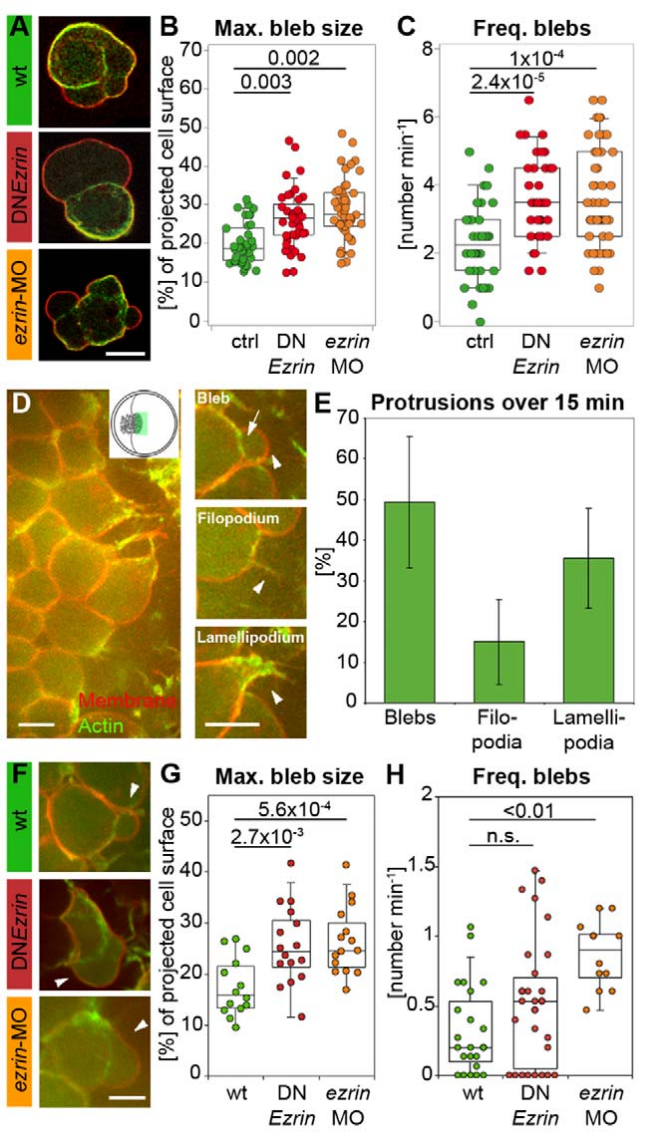
ERM proteins modulate prechordal plate progenitor cell protrusion formation. (A) Examples of isolated control and ERM-deficient prechordal plate progenitor cells. (B) Maximum bleb size in isolated control and ERM-deficient prechordal plate progenitor cells. (C) Frequency of bleb formation in isolated control and ERM-deficient prechordal plate progenitor cells. (D) Animal view of the leading edge of a wt prechordal plate. Inset shows a schematic animal view of an embryo at 80% epiboly, with the green rectangle marking the imaged area in (D). Examples of a bleb, filopodium, and lamellipodium in prechordal plate leading edge cells. Arrowheads point to the protrusions. Arrow indicates the separation between actin cortex and membrane in the bleb. (E) Percentage of blebs, filopodia, and lamellipodia in wt prechordal plate leading edge cells (mean ± half standard deviation). (F) Example blebs (arrowheads) in wt and ERM-deficient prechordal plate leading edge cells. (G) Maximum bleb size in wt and ERM-deficient prechordal plate leading edge cells. (H) Frequency of bleb formation in wt and ERM-deficient prechordal plate leading edge cells. Plasma membrane (GPI-RFP) is red; actin cortex (Lifeact-GFP) is green. Scale bars = 10 µm. The projected bleb size in (B) and (G) was normalized to the projected cell size. Number of analyzed blebs in (B) and (C) = 39 (control), 42 (DN*Ezrin*), and 51 (*ezrin*-MO) and in (G) = 14 (wt), 16 (DN*Ezrin*), and 15 (*ezrin*-MO). Number of analyzed cells in (E) and (H) = 23 (wt), 30 (DN*Ezrin*), and 12 (*ezrin*-MO). Statistical significance was determined using *t* test (G) or Mann–Whitney *U* test (B, C, and H).

Mechanical coupling of the plasma membrane to the underlying actin cortex has been proposed to influence the formation of cellular blebs [29]. Bleb-like protrusions are a common alternative to lamellipodia during migration in various cell types ranging from primordial germ cells in zebrafish to cancer cells in culture [30],[31]. We thus compared protrusion formation in isolated control and ERM-deficient prechordal plate cells expressing membrane-anchored RFP to mark the plasma membrane and Lifeact-GFP to label F-actin [32]. Isolated control cells on nonadhesive substrates formed only blebs, recognizable by the local detachment of the plasma membrane from the underlying actin cortex (Figure 2A; Video S1). Some of these blebs propagated around the cell circumference by asymmetric assembly of the actin cortex at the bleb neck, a behavior previously described as “circus movements” [33]. In contrast, ERM-deficient cells exhibited less coordinated circus movements and formed significantly larger blebs with a higher frequency (Figure 2A–2C; Videos S2 and S3). These findings indicate that reduced MCA in isolated ERM-deficient prechordal plate cells correlates with increased blebbing activity.

To determine whether similar changes in cell blebbing occur in ERM-deficient prechordal plate cells in vivo, we analyzed prechordal plate progenitor cell protrusion formation in wild type (wt) and ERM-deficient embryos expressing membrane-anchored RFP and Lifeact-GFP to distinguish between protrusion types (Videos S4, S5, S6). Three types of cellular protrusions were found in both wt and ERM-deficient prechordal plate progenitors (Figure 2D and 2E): (i) spherical protrusions initially devoid of actin, a characteristic of blebs [34], (ii) sheet-like protrusions containing actin throughout their expansion, resembling lamellipodia, and (iii) long, thin, actin-containing protrusions resembling filopodia. To quantify the formation of these different cellular protrusions in prechordal plate progenitors, we determined the frequencies of their formation, their respective proportions, and the mean time spent by the cell forming each type of protrusion. We found that in ERM-deficient prechordal plate progenitors, the frequency and size of blebs, the mean time spent blebbing, and the proportion of blebs were significantly increased, at the expense of lamellipodia and filopodia (Figures 2F–2H and S3). These observations indicate that, similar to isolated cells in culture, ERM-deficient prechordal plate progenitors with reduced MCA in vivo exhibit increased blebbing and that increased blebbing is accompanied by reduced filopodium and lamellipodium formation.

Both cortical contractility and MCA have been previously shown to be key mechanical properties controlling bleb formation [35],[36]. To exclude that changes in cortical tension rather than in MCA are responsible for the increased blebbing phenotype, we compared tension between control and ERM-deficient cells by colloidal force microscopy using AFM [37]. We found no significant differences in cell cortex tension between control and ERM-deficient cells (Figure S4), indicating that increased blebbing of ERM-deficient prechordal plate progenitors is not due to altered contractility.

**Figure 3:**
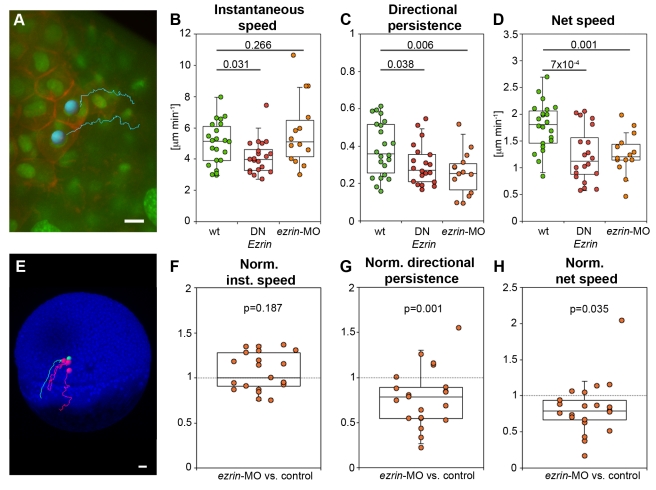
ERM proteins modulate prechordal plate progenitor cell migration. (A) Animal view of the leading edge of a wt prechordal plate with example tracks of cell nuclei movements (tracking time = 25 min). Plasma membrane (GPI-RFP) is red; nuclei (Histone-Alexa-488) are green. Scale bar = 10 µm. (B–D) Instantaneous speed (B), directional persistence (C), and net speed (D) of prechordal plate leading edge cell migration in wt and ERM-deficient embryos. (E) Lateral view of a MZ*oep* mutant embryo (blue) at 50% epiboly (6 hpf) with example tracks of control (green) and ERM-deficient mesendoderm cells (red) transplanted into the lateral germ ring margin at the onset of gastrulation (5 hpf). Tracking time = 110 min. Scale bar = 50 µm. (F–H) Instantaneous speed (F), directional persistence (G), and net speed (H) of transplanted ERM-deficient single lateral mesendoderm cells. Note that the values for speeds and directional persistence were plotted as ratios relative to transplanted control cells in the same embryo (internal controls) to reduce experimental variability between different embryos. Number of analyzed cells in (B–D) = 22 (wt), 20 (DN*Ezrin*), 13 (*ezrin*-MO), and in (F–H) = 21 *ezrin*-MO compared to control. Statistical significance was determined using *t* test (B–D) or Matlab ttest2 (F–H).

We next asked whether increased blebbing activity in ERM-deficient prechordal plate progenitors with reduced MCA changes their migratory behavior. To analyze the migratory activity of prechordal plate progenitors, we tracked the nuclei of individual progenitors at the leading edge of the prechordal plate marked with Histone-Alexa-488 from mid to late gastrulation stages (8–10 h post-fertilization [hpf]; Figure 3A; Video S7). While the instantaneous speed of the cells remained largely unchanged, we found a significant decrease in the directional persistence and thus net speed of prechordal plate progenitor cell migration in ERM-deficient embryos (Figure 3B–3D). This suggests that increased blebbing activity in ERM-deficient prechordal plate progenitors with reduced MCA leads to reduced net movement speed and directionality.

To determine whether ERM proteins function cell-autonomously in mesendoderm progenitors to modulate cell migration, we co-transplanted single mesendoderm control cells (expressing Cyc, which activates ERM proteins; [16]) with ERM-deficient cells (expressing Cyc in combination with *ezrin*-MO to inactivate ERM proteins) into the lateral side of MZ*oep* mutant embryos lacking most of their endogenous mesendoderm progenitors [38]. Under these conditions, transplanted cells only rarely interact with their neighbors and mostly undergo single cell migration [39]. We then tracked the movement of the cell nuclei from mid to late gastrulation stages (6–10 hpf; Figure 3E; Video S8). Similar to the behavior observed in the prechordal plate, transplanted ERM-deficient mesendoderm cells displayed a reduced directional persistence and slower net migration speed when compared to co-transplanted control cells, while their instantaneous speed was unchanged (Figure 3F–3H). This suggests that ERM proteins cell-autonomously modulate mesendoderm progenitor cell migration.

**Figure 4:**
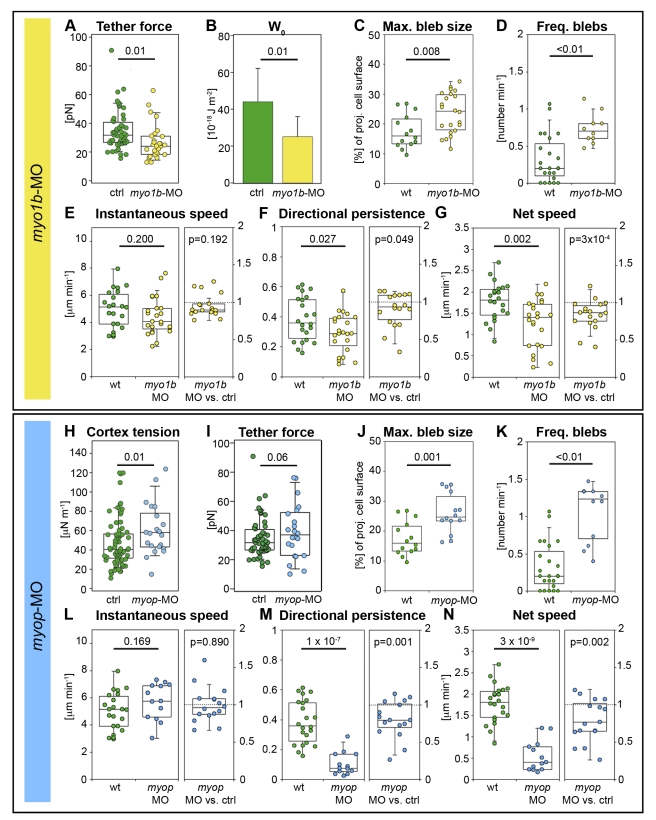
Myosin1b and Myosin phosphatase modulate prechordal plate progenitor cell protrusion formation and migration. (A) Static tether forces of isolated prechordal plate progenitor control and Myosin1b-deficient cells. (B) Adhesion energy density calculated from data presented in (A) using Equation 1. Error bars indicate absolute deviation of the median. (C) Maximum bleb size in wt and Myosin1b-deficient leading edge prechordal plate progenitor cells. (D) Frequency of bleb formation in wt and Myosin1b-deficient leading edge prechordal plate progenitor cells. (E–G) Instantaneous speed (E), directional persistence (F), and net speed (G) of prechordal plate leading edge cell migration in wt and Myosin1b-deficient embryos (left panels) and instantaneous speed, directional persistence, and net speed ratios (relative to co-transplanted control cells in the same embryo) of Myosin1b-deficient single lateral mesendoderm cells transplanted into MZ*oep* mutant embryos (right panels). (H) Cortex tension of isolated control and MyoP-deficient prechordal plate progenitor cells. (I) Static tether forces of isolated control and MyoP-deficient prechordal plate progenitor cells. (J) Maximum bleb size in wt and MyoP-deficient prechordal plate leading edge cells. (K) Frequency of bleb formation in wt and MyoP-deficient prechordal plate leading edge cells. (L–N) Instantaneous speed (L), directional persistence (M), and net speed (N) of prechordal plate leading edge cell migration in wt and MyoP-deficient embryos (left panels), and instantaneous speed, directional persistence, and net speed ratios (relative to co-transplanted control cells in the same embryo) of MyoP-deficient single lateral mesendoderm cells transplanted into MZ*oep* mutant embryos (right panels). Bleb size was normalized to cell size (C and J) as in Figure 2. Number of analyzed blebs in (C) and (J) = 14 (wt), 22 (*myo1b*-MO), and 14 (*myop*-MO). Number of analyzed cells in (D) and (K) = 23 (wt), 10 (*myo1b*-MO), and 12 (*myop*-MO); in left panels of (E–G) = 22 (wt) and 23 (*myo1b*-MO); in right panels of (E–G) = 18 *myo1b*-MO versus control; in left panels of (L–N) = 22 (wt) and 13 (*myop*-MO); and in right panels of (L–N) = 16 *myoP*-MO versus control. Statistical significance was determined using *t* test for (C), left panels of (E–G), (I), and left panels of (L–N); Mann–Whitney *U* test for (A, B, D, H, I, and K); or Matlab ttest2 for right panels of (E–G) and (L–N).

We found that in ERM-deficient cells, reduced MCA correlates with increased blebbing and that increased blebbing correlates with reduced movement directionality, which suggests that these phenotypes are functionally linked. To test whether the observed changes in cell blebbing and migration are caused by the reduction in MCA rather than by potential changes in other ERM-controlled activities, we reduced MCA independent of ERM proteins. To reduce MCA in prechordal plate progenitors, we injected a MO targeted against *myosin1b-like2* (details about MO and controls in Materials and Methods) to interfere with the activity of Myosin1b, which has been previously associated with regulating MCA [9]. Similar to ERM-deficient cells, Myosin1b-deficient mesendoderm cells exhibited reduced MCA, increased blebbing, and reduced movement directionality and net speed both within the prechordal plate and as single cells transplanted in MZ*oep* mutant embryos (Figures 4A–4G and S5; Video S9). This supports our suggestion that reducing MCA is sufficient to enhance mesendoderm cell blebbing and interfere with movement directionality and net speed, and that these phenotypes are functionally linked.

We next sought to test whether increased cell blebbing leads to the observed reduced movement directionality or whether these phenotypes are independent consequences of reduced MCA. To do so, we analyzed prechordal plate progenitor cell movement directionality when cell blebbing is increased but MCA is not reduced. To increase cell blebbing without reducing MCA, we injected a MO targeted against *myosin phosphatase*, *target subunit 2* (*myop*-MO), which has previously been shown to promote the formation of bleb-like protrusions in mesendoderm cells by activating Myosin2 ([40]; details about MO and controls in Materials and Methods). MyoP-deficient prechordal plate progenitor cells showed increased cortex tension, as well as increased blebbing activity, reduced formation of lamellipodia and filopodia, and increased MCA (Figures 4H–4K and S6; Video S10). As in ERM- and Myosin1b-deficient cells, enhanced blebbing activity of MyoP-deficient mesendoderm cells, both within the prechordal plate and as single cells transplanted in MZ*oep* mutant embryos, was accompanied by a significant reduction in the directional persistence and net speed of their migration, while the instantaneous speed of the cells did not change (Figure 4L–4N). This indicates that increased cell blebbing leads to reduced movement directionality and net speed in mesendoderm progenitors.

## Discussion

We have shown that reducing MCA in prechordal plate progenitors by interfering with the function of ERM proteins and class 1 myosins leads to increased bleb formation, at the expense of filopodia and lamellipodia, and that this increased proportion of blebs leads to less directed migration during gastrulation. These findings indicate that MCA is a key mechanical parameter controlling the protrusive and migratory activity of prechordal plate progenitor cells during gastrulation. The mechanical coupling of the plasma membrane to the underlying actin cortex has been proposed to regulate various cellular processes ranging from endocytosis to cell spreading [6]. Although MCA has been directly measured in cultured cells [6],[22],[23], very little is known about its actual regulation and function in cell morphogenesis in vivo, in particular in a developmental context. To directly evaluate the function of MCA in migrating prechordal plate progenitors in vivo, we developed a highly sensitive assay system based on AFM and high resolution confocal microscopy. We showed that changes in MCA lead to alterations in prechordal plate progenitor cell protrusion formation and migration. Moreover, to establish a causative relationship between MCA strength and prechordal plate progenitor cell morphogenesis, we showed that similar reductions in MCA due to inactivation of different proteins (ERM and Myosin1b) lead to comparable changes in cell morphogenesis. These experiments strongly support a critical function of MCA in cell protrusion formation and directed migration.

Our finding that reducing MCA in prechordal plate progenitor cells leads to an increase in the formation of blebs, as compared to lamellipodia and filopodia, suggests that MCA is an important mechanical parameter determining the proportion of different protrusion types formed by migrating cells. Decreasing MCA has previously been suggested to promote the formation of cellular blebs in cultured cells [36]; however, the mechanisms of bleb formation are still poorly understood [30]. Our finding that in both ERM- and Myosin1-deficient prechordal plate progenitors, reduced MCA leads to enhanced blebbing provides direct experimental evidence for a critical function of MCA in bleb formation during prechordal plate progenitor cell migration. MCA has also been proposed to modulate the extension of lamellipodia [7], although the role of MCA in this process in not yet clear. The observation that in Myosin1-deficient prechordal plate progenitor cells, reduced MCA increases blebbing but leaves the mean time spent forming lamellipodia unaltered (Figures 4 and S5) argues against a major function of MCA in lamellipodium formation in our system. However, as the frequency of lamellipodium formation is reduced in both ERM- and Myosin1-deficient cells (Figures S3 and S5), a role of MCA in controlling certain aspects of lamellipodium extension cannot be ruled out.

The observation that not only decreasing MCA, but also increasing cortical tension, which raises intracellular pressure, enhances blebbing in prechordal plate progenitors (Figure 4) suggests that the balance between MCA and intracellular pressure controls bleb formation. Interestingly, lowering MCA and/or elevating cortical tension increases not only the frequency but also the size of blebs (Figures 2 and 4). We have previously shown that cortical tension, and the resulting intracellular pressure, regulate bleb size by directly determining the force driving bleb expansion [35]. MCA, on the other hand, might control bleb size by regulating the size of the bleb base, which has been shown to correlate with bleb size [35] and is enlarged upon treatments reducing MCA (Figure 2). In addition, MCA might influence bleb size by setting the mechanical resistance to membrane flow into the expanding bleb, which in turn may control bleb expansion. Future studies addressing the contribution of cytoplasmic streaming, bleb base opening, and membrane flow to the dynamics of bleb growth will help to elucidate the mechanisms by which cortical tension and MCA together control bleb size and frequency.

We found that changing the proportions of blebs versus lamellipodia and filopodia by reducing MCA leads to less directed migration of prechordal plate progenitors. This finding indicates that the correct proportion of different protrusion types is critical for directed migration in these cells. Blebs are required for the directed migration of various cell types, including zebrafish primordial germ cells and cancer cells [34],[41],[42]. Studies in the teleost *Fundulus heteroclitus* have demonstrated that germ layer progenitor cells can also undergo directional migration by blebbing locomotion, suggesting that blebs are sufficient for directional migration [43],[44]. Interestingly, these cells change from bleb- to filopodium- and lamellipodium-driven migration during the course of gastrulation, resulting in individual progenitors often simultaneously forming different protrusion types [45]. While this suggests that both blebs and lamellipodia/filopodia function in directed progenitor cell migration, it remains unclear whether these different protrusion types are interchangeable or specifically contribute to directed migration. Our finding that the proportion of different protrusion types is critical for the directed migration of prechordal plate progenitors argues against interchangeability and points to specific functions for different protrusion types in this process.

Nodal/TGFβ signals are thought to be key regulators of mesendoderm cell fate specification and morphogenesis [20]. Since Nodal signaling is required for ERM phosphorylation and hence activation in mesendoderm progenitors [16], it is conceivable that Nodal proteins control mesendoderm protrusion formation and migration by regulating ERM-dependent MCA. Future studies analyzing the function of Nodal signaling in MCA will be needed to elucidate the specific contribution of MCA in Nodal-mediated mesendoderm progenitor morphogenesis.

The regulation of MCA is also likely to be important for cell migration in processes other than zebrafish gastrulation. Notably, ERM deregulation has been implicated in tumor metastasis [46], raising the possibility that the regulation of MCA is critical for cell protrusion formation and migration during tumor progression and metastasis.

## Materials and Methods

### Embryo Staging and Maintenance

Zebrafish maintenance was carried out as described in [47]. Embryos were grown at 31°C in E3 medium and staged as described in [48].

### mRNA, Morpholino, and Dye Injection

mRNA was synthesized as described in [49]. For tether force measurements wt TL embryos were injected with 100 pg of *cyc* alone (control) or together with a combination of 4 ng of *ezrin*-UTR-MO [16] plus 4 ng of *moesin-a*-MO (TGGTCTCTTCCTTCACGAATGTGTC) or 300 pg of DN*Ezrin* to generate ERM-deficient cells, 2 ng of *myop*-MO [40] to generate MyoP-deficient cells, and 8 ng of *myo1b*-UTR-MO (CGAGCAGTGATGTTTTCACCTCCAT) to generate Myo1b-deficient cells. For in vitro confocal microscopy, an additional 50 pg of *lifeact-GFP* plus 100 pg of *GPI-RFP* were injected in control and ERM-deficient embryos. For in vivo confocal microscopy, wt TL embryos were injected with 50 pg of *lifeact-GFP* plus 100 pg of *GPI-RFP* alone (control) or together with 250 pg of DN*Ezrin* (ERM-deficient), 4 ng of *ezrin*-UTR-MO (ERM-deficient), 3 ng of *myop*-MO (MyoP-deficient), or 8 ng of *myo1b*-ATG-MO (Myo1b-deficient). For tracking of prechordal plate cell nuclei, wt embryos were injected with Alexa Fluor-488 conjugated histone H1 (H13188, Invitrogen) and 100 pg of *GPI-RFP*. For tracking of cell nuclei in the transplantation experiments, wt donor embryos were injected with 100 pg of *cyc* together with Alexa Fluor-488 conjugated histone H1 (H13188, Invitrogen) (control), 100 pg of *histoneH2A-zf::mcherry* plus 4 ng of *ezrin*-UTR-MO (ERM-deficient), 100 pg of *histoneH2A-zf::mcherry* plus 3 ng of *myop*-MO [40] (MyoP-deficient), or 100 pg of *histoneH2A-zf::mcherry* plus 8 ng of *myo1b*-ATG-MO (Myo1b-deficient). MZ*oep* host embryos were injected with Dextran Alexa Fluor-647 (D22914, Invitrogen).

The *ezrin*-UTR-MO and *myop*-MO were used and controlled as described in [16]. As a further control for the *ezrin* morphant phenotype, we expressed a dominant negative non-phosphorylatable zebrafish version of *ezrin* [21], resulting in a phenotype similar to that observed in *ezrin* morphant embryos. The *myo1b*-ATG-MO was designed according to Gene Tools targeting guidelines against *myosin1b-like2* gene. To control the *myo1b* morphant phenotype, we tested a second *myo1b*-UTR-MO and a zebrafish dominant negative *myosin1b-like2* version truncated as in [50], which produced similar prechordal plate progenitor cell blebbing phenotypes as observed with the ATG-MO. We also rescued the *myo1b*-UTR-MO prechordal plate progenitor cell blebbing phenotype by co-expressing mouse full-length *myosin1a* mRNA [50] (data not shown).

### Confocal Microscopy and Bleb Size Measurement

For in vivo experiments, images were obtained with an Andor spinning disc system equipped with a 63×/1.2 objective using 488-nm and 563-nm laser lines. Frames were captured at 10-s intervals for 15 min between 8 and 10 hpf. The temperature was kept constant at 28°C. For in vitro experiments, cells from Lifeact- and GPI-RFP-expressing embryos were seeded on a BSA-coated glass slide to prevent attachment and imaged using a Leica SP5 inverted microscope equipped with a 63×/1.2 lens using 488-nm and 561-nm laser lines for 2 min at 2-s intervals. For bleb size measurements, the projected area of the bleb at its maximal extension was measured using ImageJ and normalized to the projected area of the whole cell.

### Transplantation Experiments

Wt TL and MZ*oep* mutant donor and host embryos were dechorionated with Pronase (2 mg·ml^−1^ in E2) and transferred onto an agarose plate with E3 medium. Two to three cells were taken from control and experimental donor embryos at dome stage (5 hpf) and transplanted into the emerging lateral mesendoderm of a MZ*oep* dharma::GFP host embryo labeled with Dextran Alexa Fluor-647 at shield stage (6 hpf). Time-lapse images were obtained with an upright Leica SP5 confocal microscope equipped with a 20× water immersion lens using 488-nm Argon, DPSS 561-nm, and 633-nm HeNe laser lines. Frames were captured at 90-s intervals for 3.5 h (7–10 hpf). The temperature was kept constant in all videos (28°C).

### Cell Tracking

Cell/nuclei tracking in three dimensions (*x*, *y*, and *z*) was performed with Imaris 6.2.0 software. The instantaneous and net speeds, as well as directional persistence (ratio of the net displacement to the distance actually traveled by the cells), were extracted from the tracks.

### Tether Extrusion Using Atomic Force Microscopy

Tethers were extruded as described in [24] using a JPK Instruments Nanowizard equipped with a CellHesion module. In short, Olympus Biolevers (*k* = 6 mN·m^−1^) were plasma-cleaned and incubated in 2.5 mg·ml^−1^ Concanavalin A (Sigma) for 4 h at room temperature. Before the measurements, cantilevers were rinsed in PBS plus Ca^2+^ and calibrated using the thermal noise method. For the measurement, cells were seeded on a glass slide in a home-built fluid chamber filled with DMEM-F12 cell culture medium and not used longer than 1 h for data acquisition. To depolymerize actin, cells were treated with 1 µM LatA for 10 min. Approach velocity was set to 5 µm·s^−1^, contact time was minimized to yield an interaction in 30% of all contacts (between 0.0 and 0.6 s), and contact force was set to 100 pN. For static tether force measurements, the cantilever was retracted for 6 µm at a speed of 10 µm·s^−1^, and the position was kept constant for 30 s. Resulting force–time curves were analyzed using IgorPro. For dynamic tether force measurements, each cell was probed with different speeds ranging from 1 to 50 µm·s^−1^ in a random order. Tethers were allowed to retract completely between successive pulls. Raw data were analyzed using a home-written IgorPro procedure adapted from the Kerssemakers algorithm.

### Tether Data Analysis and Model Assumptions

Static and dynamic tether pulling experiments were used to measure the MCA energy density *W*_0_ and the density of plasma-membrane-to-cortex cross-linking molecules ν, respectively. For static tether pulling, Equation 1, described in the Results, was used to extract *W*_0_ from the static tether force (*F*_0_). For dynamic tether pulling experiments, the force (*f*)–velocity (*ν*) profiles were analyzed using the model described in [23], where the pulling force depends on the surface viscosity of the plasma membrane η and on ν:
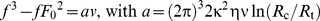
(2)where *F*_0_ is the static tether force, *R*_c_ is the radius of the cell, and *R*_t_ is the radius of the tether. The model was fitted to the data using a home-written least squares minimization procedure. This yielded the static tether force *F*_0_ and the coefficient characterizing the dynamics of extrusion, *a*. Values for cell radius were measured with light microscopy (Figure S2H), and the tether radius was calculated from static tether forces according to *R*_t_ = 2πκ/*F*_0_ [22].

The other parameters of the model (Equations 1 and 2) are the plasma membrane bending rigidity κ, the membrane tension σ, and the membrane surface viscosity η. All three are properties of the plasma membrane that change only if the composition of the membrane itself changes, which is unlikely to happen upon perturbations affecting proteins lying within the cortex under the plasma membrane [51]. Supporting this assumption, FRAP experiments showed that the diffusion coefficient of lipids within the plasma membrane was not changed between ERM-deficient and control cells (Figure S2A–S2F; for methods see Text S1), suggesting that membrane surface viscosity η was unchanged. Moreover, we measured the tether force (*F*) and membrane tension (σ) in isolated control and ERM-deficient prechordal plate progenitor cells that were treated with LatA to disassemble their actin cortex. The tether force in LatA-treated cells is determined by σ and κ only (see also Equation 1). Both the tether force *F* and the membrane tension σ remained unchanged in LatA-treated ERM-deficient cells relative to control LatA-treated cells (Table S1 and data not shown), suggesting that κ is also unchanged. The values of κ, σ, and η were thus kept constant for all the experimental conditions. κ and η were taken from the literature with κ = 2.9×10^−19^ N•m [22],[25],[36] and η = 1.5×10^−7^ Pa•m•s [26]. Plasma membrane tension σ was calculated from tether pulling experiments using cells treated with LatA (*T*_app_ = σ = 2.5 µN•m^−1^). During tether extrusion, the model assumes that the lipids flow past the cytoskeleton-bound transmembrane molecules as they are dragged into the tether (permeation regime). This is true for intermediate velocities up to several 100 µm•s^−1^ (Figure S2I), while transmembrane molecules unbind from the cortical cytoskeleton if tethers are extruded faster or membrane viscosity becomes greater [23]. Since the tether pulling velocities in our experiments were ≤50 µm•s^−1^, we were most likely within the permeation regime, allowing us to investigate the density of binding molecules. No history effect was observed when sequential tethers were extruded from one cell (Figure S2J).

### Cortex Tension Measurements by Colloidal Force Microscopy

Cortex tension measurements were carried out as described previously [37]. In short, an AFM cantilever was modified with a glass bead (diameter *D* = 5 µm) and coated with heat-inactivated FCS to prevent unspecific binding with the cell during the contact measurement. The colloidal force probe was then brought into contact with the cell with 500 pN contact force at 1 µm•s^−1^. A fit to the cortical shell liquid core model [37] between 125 pN and 250 pN yielded cortex tension. To depolymerize actin, cells were treated with 1 µM LatA for 10 min.

### Statistical Analysis

Analysis of variance (ANOVA) and *t* tests were performed after data were confirmed to have normal distribution and equal variance; otherwise, Kruskal–Wallis tests or Mann–Whitney *U* tests were applied. *p*-values were computed in R. For cell transplantation experiments, ttest2 from Matlab was used, which compared our data points with a random distribution of numbers around one with the same standard deviation as our data.

## Supporting Information

Figure S1 **ERM-deficient embryos show reduced convergence and extension movements during gastrulation.** (A) Quantification of prechordal plate width-to-length ratio (normalized to wt) in embryos expressing either GPI-RFP and Lifeact-GFP alone (control) or together with DN*Ezrin* (250 pg), DN*Ezrin* (500 pg), or *ezrin*-MO (4 ng) at the bud stage (10 hpf) stained for *notail* (*ntl*) marking the notochord, *distal-less homeobox 3* (*dlx3*) marking the anterior edge of the neural plate, and *hatching gland gene-1* (*hgg1*) marking the prechordal plate. (B) Quantification of notochord length (normalized to wt) of control and experimental embryos. *p*-values were calculated using *t* test. Pictures are representative examples of wt and morphant embryos stained in situ. For methods see Text S1.
(0.61 MB TIF)

Figure S2 **Physical properties of the plasma membrane and cortex in control and ERM-deficient prechordal plate progenitor cells.** (A–F) Plasma membrane fluidity measurements of control and ERM-deficient prechordal plate progenitor cells. Sequential images of a typical FRAP experiment before bleaching (A), directly after bleaching (B), and after complete recovery (C). Red square demarcates bleached region. (D) Kymograph of the bleached region. The kymograph was calculated on a line (width = one pixel) encompassing the bleached region of the cell membrane within the red box. Recovery occurs from the rims of the bleached area. Scale bars: in *y* = 5 µm and in *x* = 9 s. (E) Example of a recovery curve for a prechordal plate progenitor cell expressing GAP43-GFP. (F) Diffusion coefficient extracted from FRAP experiments in control, ERM-deficient, and LatA-treated prechordal plate progenitor cells. For more details see Text S1. (G) Average lateral separation between plasma-membrane-to-cortex cross-linking molecules of control, ERM-deficient, and LatA-treated prechordal plate progenitor cells. Error bars indicate error propagated from the fit. (H) Cell radius measured during cortical tension measurements. Error bars indicate error propagated from the fit. *p*-value was estimated using *t* test. (I) Estimation of the range of velocities for which Equation 2 is valid. The plot represents theoretical curves, computed as in [52], of the forces exerted on transmembrane proteins during tether extraction. The friction force (*F*_f_, dark grey squares) due to the flow of membrane into the tether increases linearly with pulling velocities, whereas the rupture force (*F*_r_, light grey squares), at which the transmembrane protein would unbind from the cortex, increases logarithmically. The intersection between these two curves (600 µm·s^−1^) gives the critical velocity at which transmembrane proteins unbind from the cortex. Therefore, the velocities we used for tether pulling (lower than 100 µm·s^−1^) are suitable to estimate ν. The parameters used to compute the curves are the membrane viscosity (1.5×10^−7^ Pa•m•s; see Materials and Methods) and the typical distance between cortex–membrane linkers (0.35 nm [53]). (J) Comparison between the extrusion force of the first tether that has been extruded from a cell and the tether force of all measurements shows that force is not influenced by the pulling history. The fit parameters are *a* = 9,969 and *F*_0_ = 32 pN for “all curves” and *a* = 11,612 and *F*_0_ = 25 pN for “first curves.”
(0.98 MB TIF)

Figure S3 **ERM proteins modulate protrusion formation in prechordal plate progenitors.** (A, D, and G) Histograms of proportion of blebs (A), lamellipodia (D), and filopodia (G) in wt and ERM-deficient prechordal plate leading edge cells (*p*<0.05 for blebs and *p*>0.05 for lamellipodia and filopodia in DN*Ezrin* embryos, and *p*<0.01 for all three types of protrusions in *ezrin*-MO morphant embryos; bin size = 10%). (B, E, and H) Mean time spent blebbing (B), forming lamellipodia (E), or forming filopodia (H) in wt and ERM-deficient prechordal plate leading edge cells within a 15-min time interval. (C and F) Frequency of lamellipodium (C) and filopodium (F) formation in wt and ERM-deficient prechordal plate leading edge cells. Statistical significance was determined using Mann–Whitney *U* test (A, C, D, F, and G) and *t* test (B, E, and H). Number of analyzed cells = 23 (wt), 30 (DN*Ezrin*), and 12 (*ezrin*-MO).
(0.86 MB TIF)

Figure S4 **Cortex tension is unaffected in ERM-deficient prechordal plate progenitor cells.** (A) Schematic outline of the experiment. The indentation of a cell is monitored after a bead-coupled AFM cantilever is brought into contact with a weakly adherent cell on a substrate applying a predefined force. (B) Example force–distance curves of control and LatA-treated cells. (C) Cortical tension (*T*_c_) for control, ERM-deficient, and LatA-treated cells. Number of analyzed cells = 91 (control), 28 (MO), 88 (DN*Ezrin*), and 32 (LatA).
(0.35 MB TIF)

Figure S5 **Myosin1b modulates protrusion formation in prechordal plate progenitors.** (A, D, and G) Histograms of proportion of blebs (A), lamellipodia (D), and filopodia (G) in wt and Myosin1b-deficient prechordal plate leading edge cells (*p*<0.01 for all three protrusion types; bin size = 10%). (B, E, and H) Mean time spent blebbing (B), forming lamellipodia (E), or forming filopodia (H) in wt and Myosin1b-deficient prechordal plate leading edge cells within a 15-min time interval. (C and F) Frequency of lamellipodium (C) and filopodium (F) formation in wt and Myosin1b-deficient prechordal plate leading edge cells. Statistical significance was determined using Mann–Whitney *U* test (A, C, D, F, and G) and *t* test (B, E, and H). Number of analyzed cells = 23 (wt) and 10 (*myo1b*-MO).
(0.69 MB TIF)

Figure S6 **Myosin phosphatase modulates protrusion formation in prechordal plate progenitors.** (A, D, and G) Histograms of proportion of blebs (A), lamellipodia (D), and filopodia (G) in wt and MyoP-deficient prechordal plate leading edge cells (*p*<0.01 for all three protrusion types; bin size = 10%). (B, E, and H) Mean time spent blebbing (B), forming lamellipodia (E), or forming filopodia (H) in wt and MyoP-deficient prechordal plate leading edge cells within a 15-min time interval. (C and F) Frequency of lamellipodium (C) and filopodium (F) formation in wt and MyoP-deficient prechordal plate leading edge cells. Statistical significance was determined using Mann–Whitney *U* test (A, C, D, F, and G) and *t* test (B, E, and H). Number of analyzed cells = 23 (wt) and 12 (*myop*-MO).
(0.63 MB TIF)

Table S1 **Statistics and comparisons for static and dynamic tether force measurements.** (A) Number of tethers pulled and average tether forces for the dynamic tether pulling experiments (Figure 1E–1H). (B) Number of tethers pulled and median tether forces probed for the height-clamp experiments (Figure 1A–1D). Last column are values of *F*_0_ extracted from the fit of Equation 2 to the force–velocity data in Figure 1G.
(0.19 MB PDF)

Text S1 **Materials and methods used for supporting figures.**
(0.06 MB DOC)

Video S1 **Bleb formation in isolated prechordal plate progenitor cells.** Control prechordal plate progenitor cell expressing GPI-RFP and Lifeact-GFP and imaged for 2 min on a BSA-coated glass slide. Frame rate = 2 s; scale bar = 10 µm.
(1.31 MB MOV)

Video S2 **Bleb formation is enhanced in isolated DN** ***Ezrin*** **-expressing prechordal plate progenitor cells.** ERM-deficient (DN*Ezrin*-expressing) prechordal plate progenitor cell expressing GPI-RFP and Lifeact-GFP imaged for 2 min on a BSA-coated glass slide. Frame rate = 2 s; scale bar = 10 µm.
(0.62 MB MOV)

Video S3 **Bleb formation is enhanced in isolated** ***ezrin/moesin-a*** **morphant prechordal plate progenitor cells.** ERM-deficient prechordal plate progenitor cell (injected with *ezrin*-MO and *moesin-a*-MO) and expressing GPI-RFP and Lifeact-GFP imaged for 2 min on a BSA-coated glass slide. Frame rate = 2 s; scale bar = 10 µm.
(1.08 MB MOV)

Video S4 **Protrusion formation and migration of wt prechordal plate progenitors.** Animal view of the leading edge of the prechordal plate of a wt embryo. Plasma membrane (GPI-RFP) is red; actin cortex (Lifeact-GFP) is green. Scale bar = 10 µm. Time in minutes:seconds.
(3.52 MB MOV)

Video S5 **Bleb formation is enhanced in DN** ***Ezrin*** **-expressing prechordal plate progenitors.** Animal view of the leading edge of the prechordal plate of an embryo expressing DN*Ezrin*. Plasma membrane (GPI-RFP) is red; actin cortex (Lifeact-GFP) is green. Scale bar = 10 µm. Time in minutes:seconds.
(2.45 MB MOV)

Video S6 **Bleb formation is enhanced in** ***ezrin*** **morphant prechordal plate progenitors.** Animal view of the leading edge of the prechordal plate of an embryo injected with *ezrin*-MO. Plasma membrane (GPI-RFP) is red; actin cortex (Lifeact-GFP) is green. Scale bar = 10 µm. Time in minutes:seconds.
(2.67 MB MOV)

Video S7 **Tracking of prechordal plate progenitor cell migration.** Animal view of the leading edge of the prechordal plate of a wt embryo. Blue dots (marking nuclei) are tracked with Imaris. Plasma membrane (GPI-RFP) is red; nuclei (Histone-Alexa-488) are green. Scale bar = 10 µm. Time in minutes:seconds.
(2.07 MB MOV)

Video S8 **ERM proteins cell-autonomously modulate prechordal plate progenitor cell migration.** Lateral view of a MZ*oep* mutant embryo in which control and ERM-deficient prechordal plate progenitors were co-transplanted at 50% epiboly (5 hpf). Nuclei were tracked with Imaris from mid to late gastrulation stages (6–10 hpf). Control cells are green; ERM-deficient cells are red. Scale bar = 50 µm. Time in minutes:seconds.
(4.71 MB MOV)

Video S9 **Bleb formation is enhanced in** ***myo1b*** **morphant prechordal plate progenitors.** Animal view of the leading edge of the prechordal plate of an embryo injected with *myo1b*-MO. Plasma membrane (GPI-RFP) is red; actin cortex (Lifeact-GFP) is green. Scale bar = 10 µm. Time in minutes:seconds.
(1.64 MB MOV)

Video S10 **Bleb formation is enhanced in** ***myop*** **morphant prechordal plate progenitors.** Animal view of the leading edge of the prechordal plate of an embryo injected with *myop*-MO. Plasma membrane (GPI-RFP) is red; actin cortex (Lifeact-GFP) is green. Scale bar = 10 µm. Time in minutes:seconds.
(2.63 MB MOV)

## Abbreviations

AFM: atomic force microscopy
ERM: Ezrin/Radixin/Moesin
FRAP: fluorescence recovery after photobleaching
hpf: hours post-fertilization
LatA: Latrunculin A
MCA: membrane-to-cortex attachment
MO: morpholino
wt: wild-type
