# Supplementary figures and images for: Control of Directed Cell Migration In Vivo by Membrane-to-Cortex Attachment
Source: PLoS Biol. 2010 Nov 30;8(11):e1000544. doi: 10.1371/journal.pbio.1000544 (PMC2994655; doi:10.1371/journal.pbio.1000544)

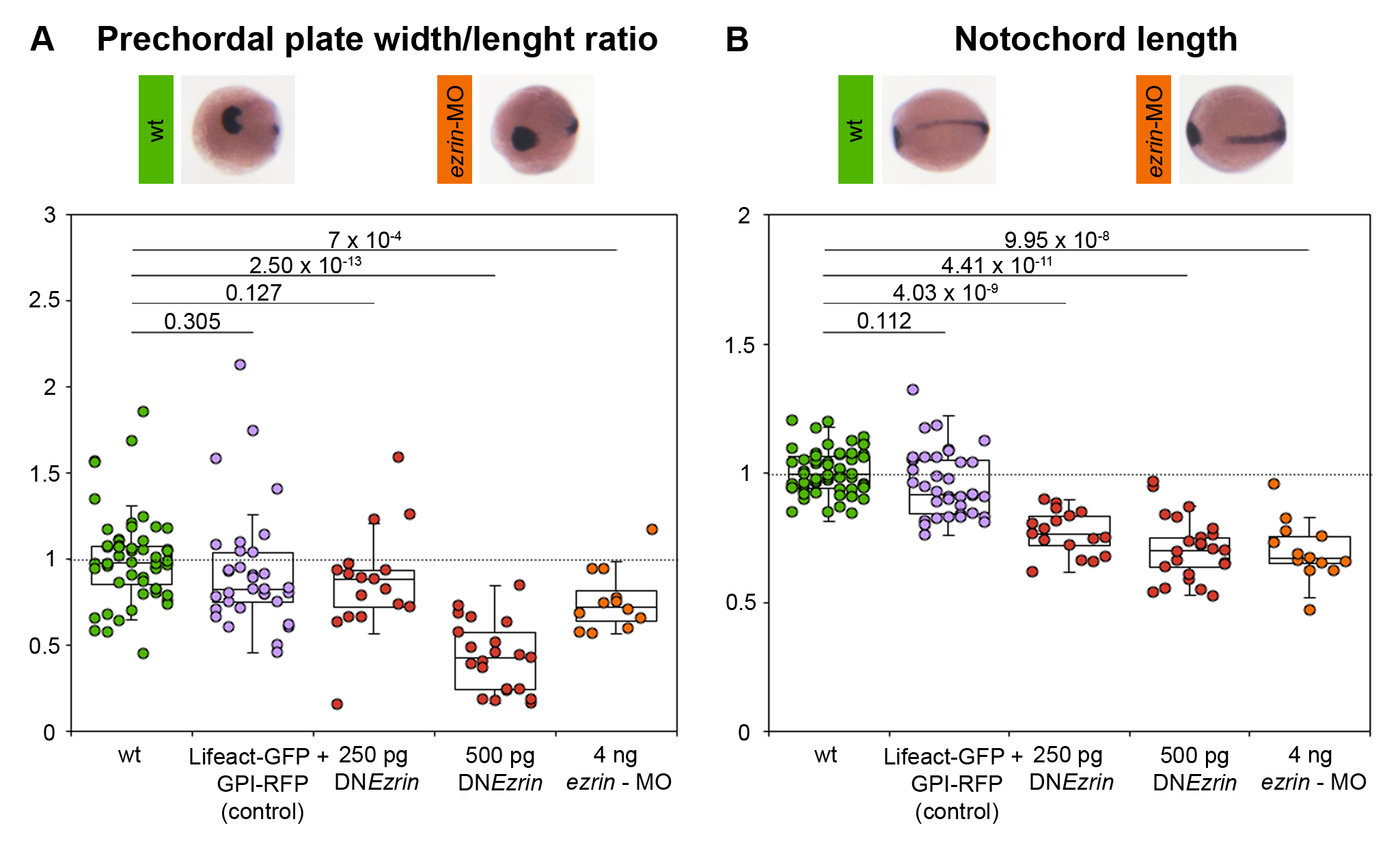

Supplement: Figure S1 — ERM-deficient embryos show reduced convergence and extension movements during gastrulation. (A) Quantification of prechordal plate width-to-length ratio (normalized to wt) in embryos expressing either GPI-RFP and Lifeact-GFP alone (control) or together with DNEzrin (250 pg), DNEzrin (500 pg), or ezrin-MO (4 ng) at the bud stage (10 hpf) stained for notail (ntl) marking the notochord, distal-less homeobox 3 (dlx3) marking the anterior edge of the neural plate, and hatching gland gene-1 (hgg1) marking the prechordal plate. (B) Quantification of notochord length (normalized to wt) of control and experimental embryos. p-values were calculated using t test. Pictures are representative examples of wt and morphant embryos stained in situ. For methods see Text S1. (0.61 MB TIF) [file pbio.1000544.s001.tif]

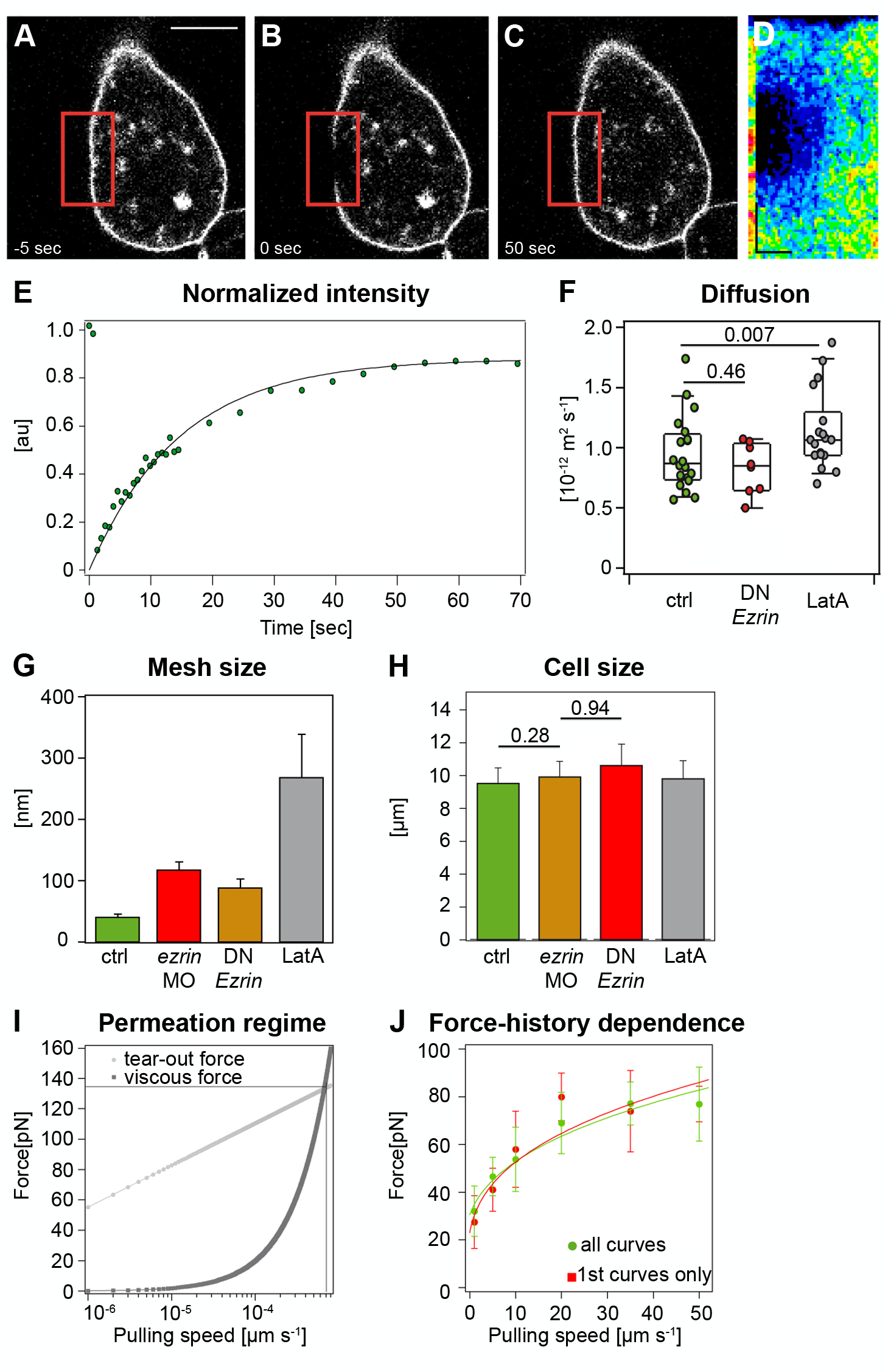

Supplement: Figure S2 — Physical properties of the plasma membrane and cortex in control and ERM-deficient prechordal plate progenitor cells. (A–F) Plasma membrane fluidity measurements of control and ERM-deficient prechordal plate progenitor cells. Sequential images of a typical FRAP experiment before bleaching (A), directly after bleaching (B), and after complete recovery (C). Red square demarcates bleached region. (D) Kymograph of the bleached region. The kymograph was calculated on a line (width = one pixel) encompassing the bleached region of the cell membrane within the red box. Recovery occurs from the rims of the bleached area. Scale bars: in y = 5 µm and in x = 9 s. (E) Example of a recovery curve for a prechordal plate progenitor cell expressing GAP43-GFP. (F) Diffusion coefficient extracted from FRAP experiments in control, ERM-deficient, and LatA-treated prechordal plate progenitor cells. For more details see Text S1. (G) Average lateral separation between plasma-membrane-to-cortex cross-linking molecules of control, ERM-deficient, and LatA-treated prechordal plate progenitor cells. Error bars indicate error propagated from the fit. (H) Cell radius measured during cortical tension measurements. Error bars indicate error propagated from the fit. p-value was estimated using t test. (I) Estimation of the range of velocities for which Equation 2 is valid. The plot represents theoretical curves, computed as in [52], of the forces exerted on transmembrane proteins during tether extraction. The friction force (F f, dark grey squares) due to the flow of membrane into the tether increases linearly with pulling velocities, whereas the rupture force (F r, light grey squares), at which the transmembrane protein would unbind from the cortex, increases logarithmically. The intersection between these two curves (600 µm·s−1) gives the critical velocity at which transmembrane proteins unbind from the cortex. Therefore, the velocities we used for tether pulling (lower than 100 µm·s−1) are suitab [file pbio.1000544.s002.tif]

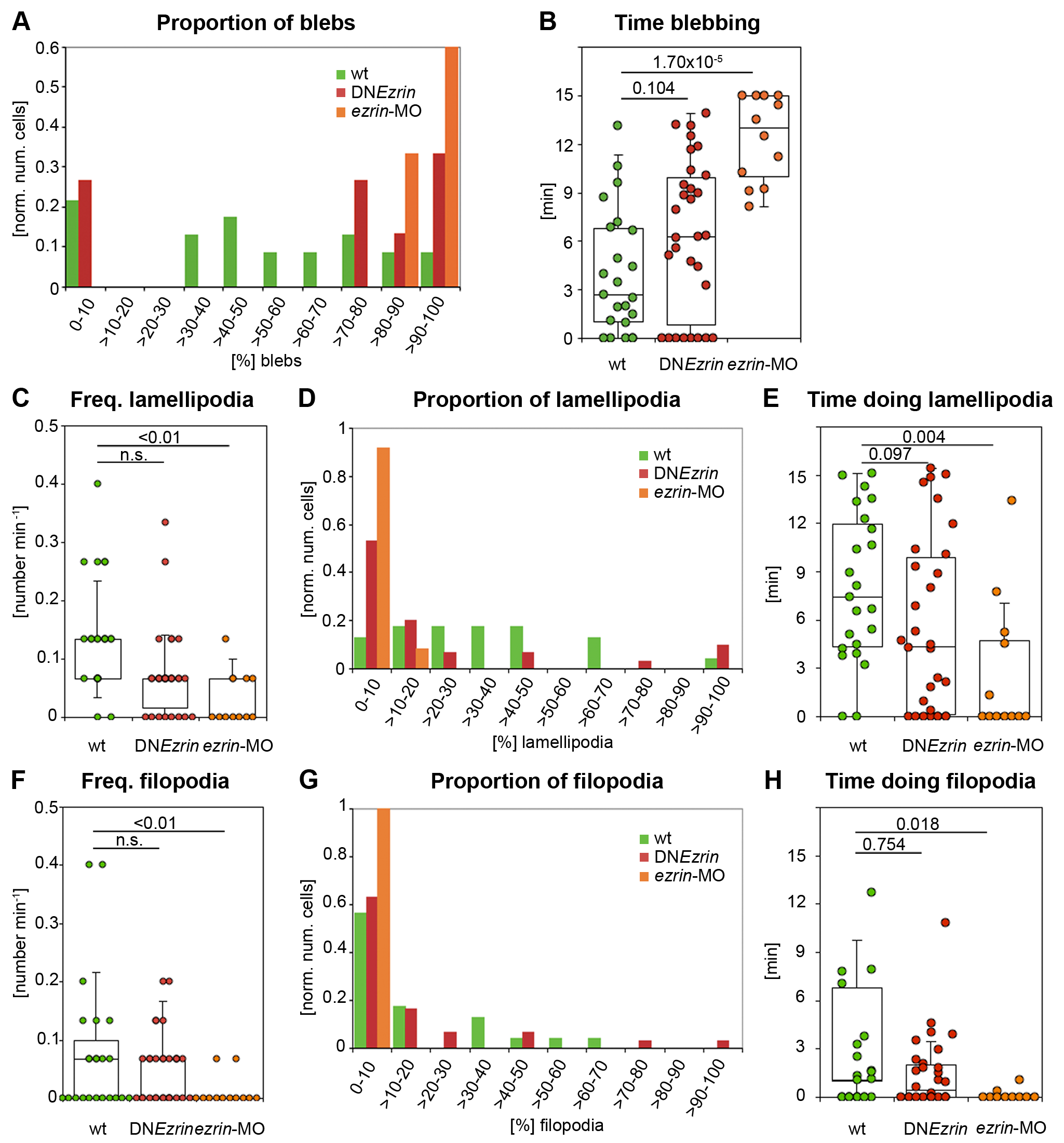

Supplement: Figure S3 — ERM proteins modulate protrusion formation in prechordal plate progenitors. (A, D, and G) Histograms of proportion of blebs (A), lamellipodia (D), and filopodia (G) in wt and ERM-deficient prechordal plate leading edge cells (p<0.05 for blebs and p>0.05 for lamellipodia and filopodia in DNEzrin embryos, and p<0.01 for all three types of protrusions in ezrin-MO morphant embryos; bin size = 10%). (B, E, and H) Mean time spent blebbing (B), forming lamellipodia (E), or forming filopodia (H) in wt and ERM-deficient prechordal plate leading edge cells within a 15-min time interval. (C and F) Frequency of lamellipodium (C) and filopodium (F) formation in wt and ERM-deficient prechordal plate leading edge cells. Statistical significance was determined using Mann–Whitney U test (A, C, D, F, and G) and t test (B, E, and H). Number of analyzed cells = 23 (wt), 30 (DNEzrin), and 12 (ezrin-MO). (0.86 MB TIF) [file pbio.1000544.s003.tif]

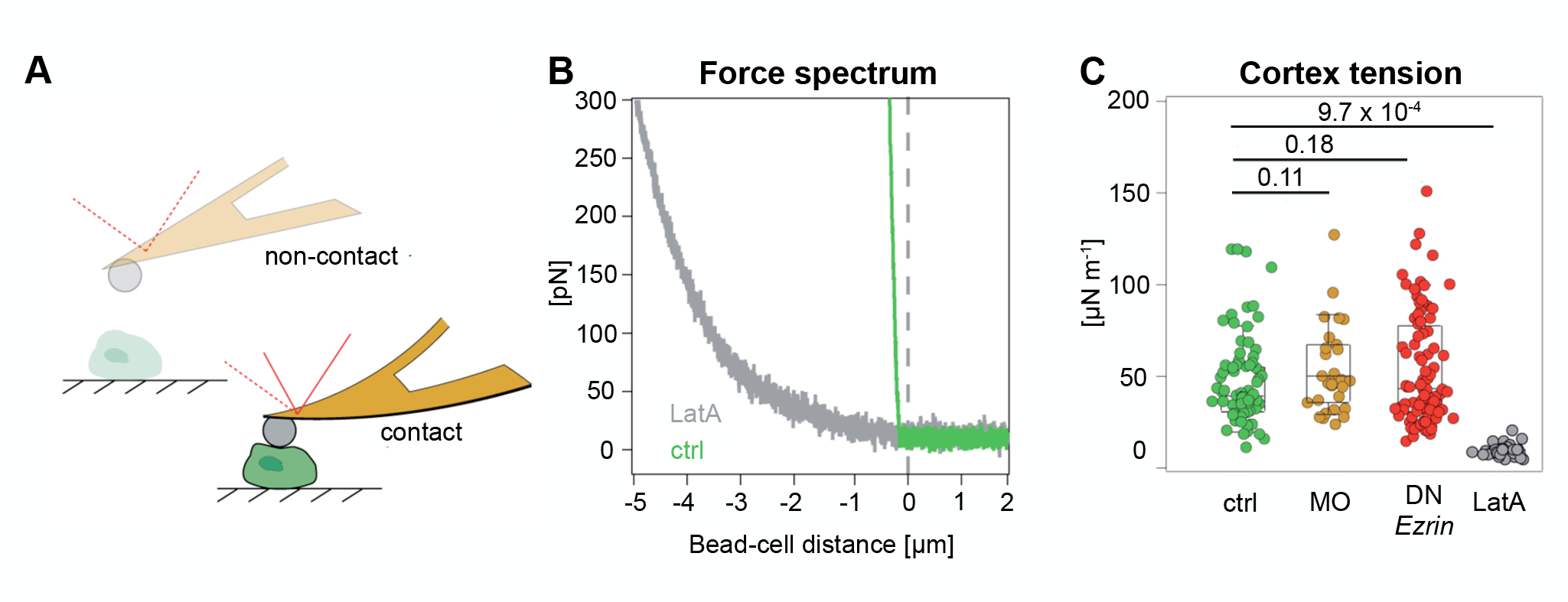

Supplement: Figure S4 — Cortex tension is unaffected in ERM-deficient prechordal plate progenitor cells. (A) Schematic outline of the experiment. The indentation of a cell is monitored after a bead-coupled AFM cantilever is brought into contact with a weakly adherent cell on a substrate applying a predefined force. (B) Example force–distance curves of control and LatA-treated cells. (C) Cortical tension (T c) for control, ERM-deficient, and LatA-treated cells. Number of analyzed cells = 91 (control), 28 (MO), 88 (DNEzrin), and 32 (LatA). (0.35 MB TIF) [file pbio.1000544.s004.tif]

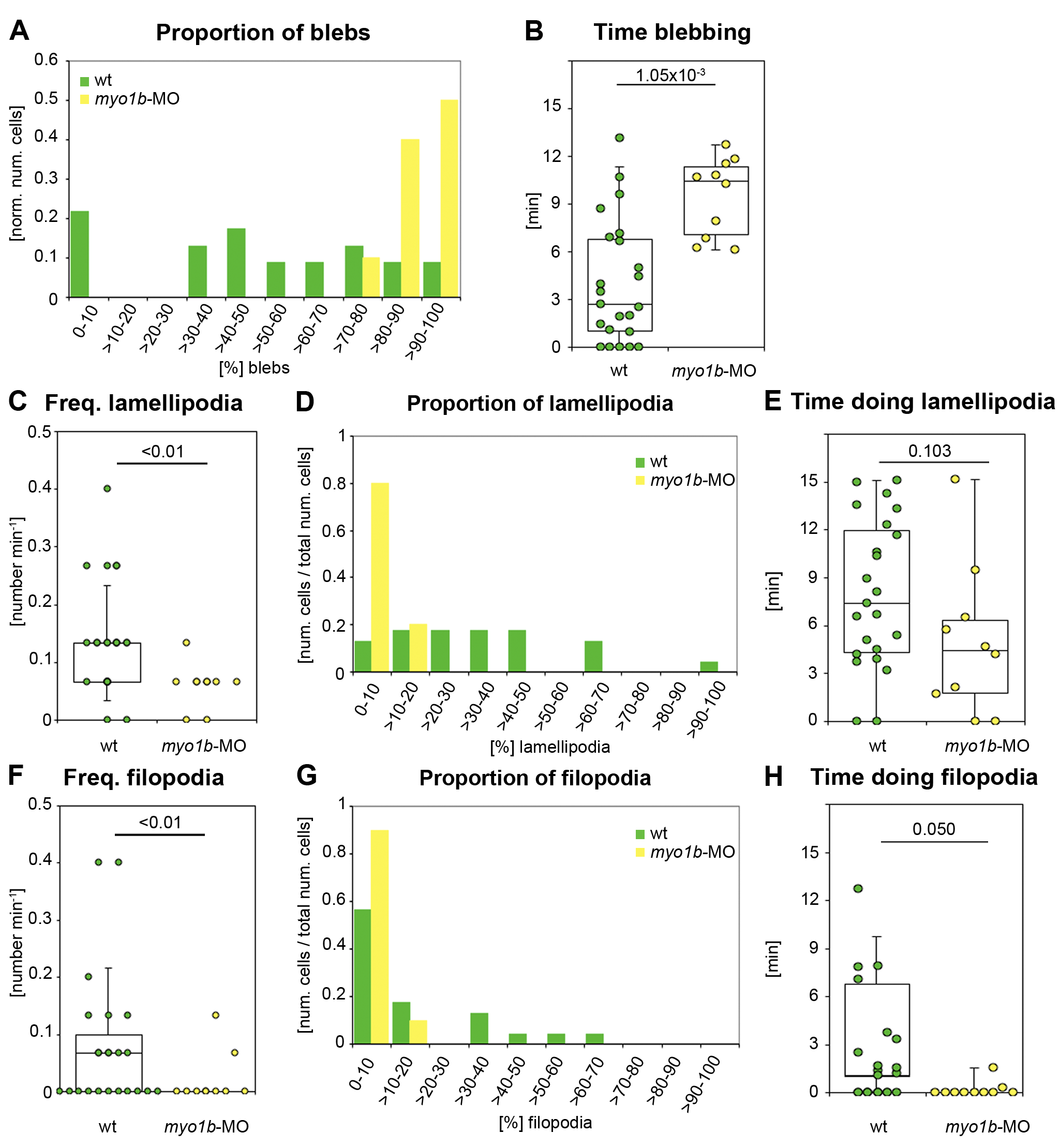

Supplement: Figure S5 — Myosin1b modulates protrusion formation in prechordal plate progenitors. (A, D, and G) Histograms of proportion of blebs (A), lamellipodia (D), and filopodia (G) in wt and Myosin1b-deficient prechordal plate leading edge cells (p<0.01 for all three protrusion types; bin size = 10%). (B, E, and H) Mean time spent blebbing (B), forming lamellipodia (E), or forming filopodia (H) in wt and Myosin1b-deficient prechordal plate leading edge cells within a 15-min time interval. (C and F) Frequency of lamellipodium (C) and filopodium (F) formation in wt and Myosin1b-deficient prechordal plate leading edge cells. Statistical significance was determined using Mann–Whitney U test (A, C, D, F, and G) and t test (B, E, and H). Number of analyzed cells = 23 (wt) and 10 (myo1b-MO). (0.69 MB TIF) [file pbio.1000544.s005.tif]

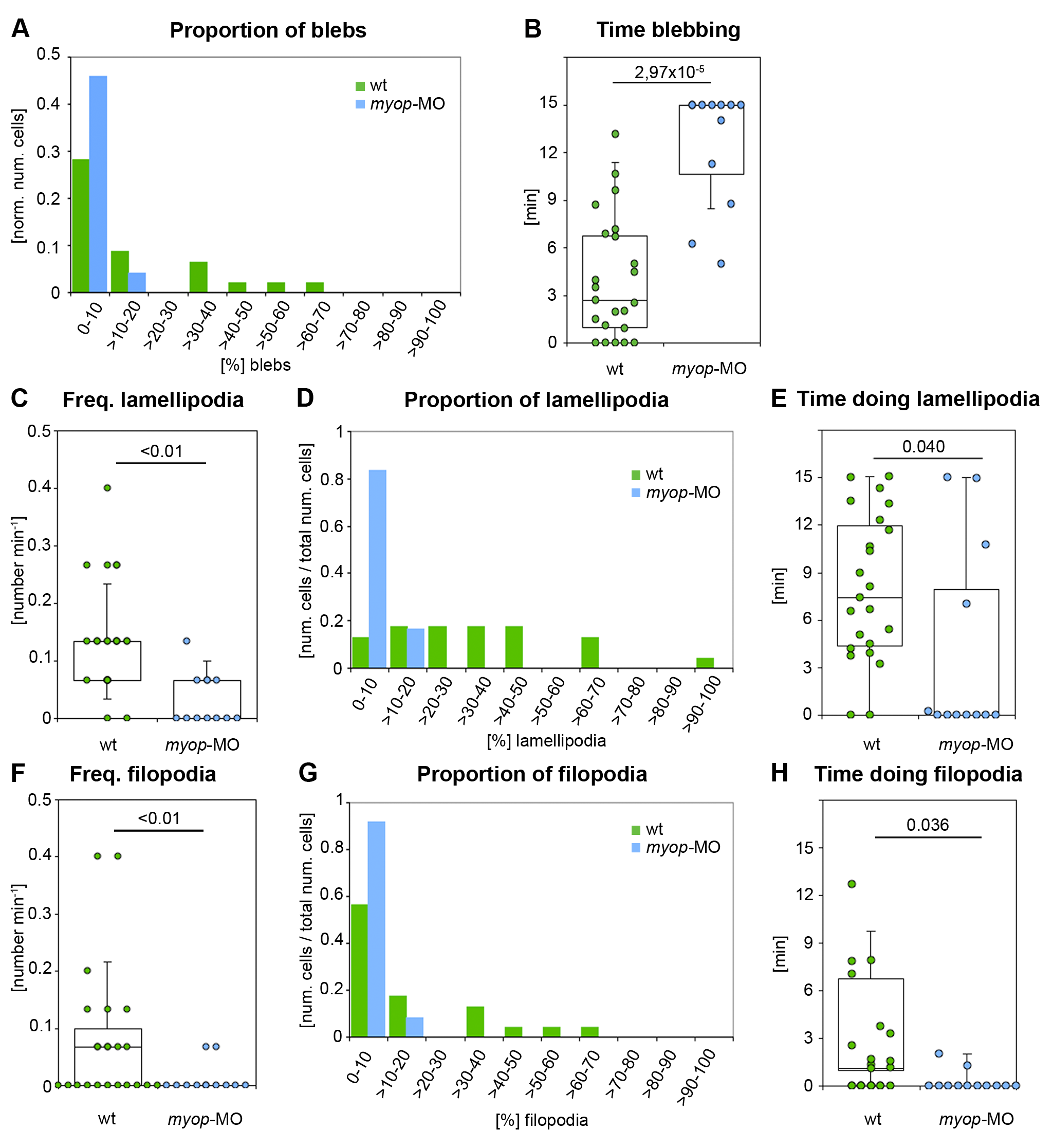

Supplement: Figure S6 — Myosin phosphatase modulates protrusion formation in prechordal plate progenitors. (A, D, and G) Histograms of proportion of blebs (A), lamellipodia (D), and filopodia (G) in wt and MyoP-deficient prechordal plate leading edge cells (p<0.01 for all three protrusion types; bin size = 10%). (B, E, and H) Mean time spent blebbing (B), forming lamellipodia (E), or forming filopodia (H) in wt and MyoP-deficient prechordal plate leading edge cells within a 15-min time interval. (C and F) Frequency of lamellipodium (C) and filopodium (F) formation in wt and MyoP-deficient prechordal plate leading edge cells. Statistical significance was determined using Mann–Whitney U test (A, C, D, F, and G) and t test (B, E, and H). Number of analyzed cells = 23 (wt) and 12 (myop-MO). (0.63 MB TIF) [file pbio.1000544.s006.tif]
