# Supplementary material for: Control of Directed Cell Migration In Vivo by Membrane-to-Cortex Attachment
Source: PLoS Biol. 2010 Nov 30;8(11):e1000544. doi: 10.1371/journal.pbio.1000544 (PMC2994655; doi:10.1371/journal.pbio.1000544)
Supplement: Table S1 — Statistics and comparisons for static and dynamic tether force measurements. (A) Number of tethers pulled and average tether forces for the dynamic tether pulling experiments (Figure 1E–1H). (B) Number of tethers pulled and median tether forces probed for the height-clamp experiments (Figure 1A–1D). Last column are values of F 0 extracted from the fit of Equation 2 to the force–velocity data in Figure 1G. (0.19 MB PDF) [file pbio.1000544.s007.pdf]

A

	$1\ \mu m \cdot s^{-1}$		$5\ \mu m \cdot s^{-1}$		$10\ \mu m \cdot s^{-1}$	
cell type	mean	$N_{tethers}$ (cells)	mean	$N_{tethers}$ (cells)	mean	$N_{tethers}$ (cells)
Control	41.5pN	94(42)	51.2pN	101(40)	56.7pN	137(38)
+DN*Ezrin*	22pN	38(22)	28.8pN	50(26)	30.3pN	70(30)
+*ezrin2, radixin* MO	29.1pN	77(37)	32.8pN	112(37)	39pN	130(37)
+Latrunculin A (LatA)	14.6pN	58(28)	20.5pN	72(29)	19pN	81(34)
+DN*Ezrin*+LatA	12pN	28(13)	16.6pN	29(15)	19.3pN	31(15)

	$20\ \mu m \cdot s^{-1}$		$35\ \mu m \cdot s^{-1}$		$50\ \mu m \cdot s^{-1}$	
cell type	mean	$N_{tethers}$ (cells)	mean	$N_{tethers}$ (cells)	mean	$N_{tethers}$ (cells)
Control	67pN	116(39)	76pN	26(13)	79pN	68(29)
+DN*Ezrin*	33pN	50(26)	39.6pN	14(6)	44pN	66(27)
+*ezrin2, radixin* MO	42pN	142(40)	47pN	40(16)	48.5pN	55(20)
+LatA	22.9pN	71(24)	21.4pN	24(12)	23.7pN	47(20)
+DN*Ezrin*+LatA	23pN	28(12)	28pN	21(10)	32.5pN	15(10)

B

cell type	$\mathbf{F}_0^{Median}$	$N_{tethers}$ (cells)	$\mathbf{F}_0^{fit}$
Control	32pN	114(56)	**32.3pN**
+DN*Ezrin*	19.1pN	48(24)	**19.1pN**
+*ezrin2, radixin* MO	17pN	76(45)	**25pN**
+LatA	9.9pN	9(5)	**15.1pN**
+*myo1b* MO	24pN	49(31)	-
+*myoPhosphatase* MO	41.8pN	45(22)	-
