# Supplementary material for: Control of Directed Cell Migration In Vivo by Membrane-to-Cortex Attachment
Source: PLoS Biol. 2010 Nov 30;8(11):e1000544. doi: 10.1371/journal.pbio.1000544 (PMC2994655; doi:10.1371/journal.pbio.1000544)
Supplement: Text S1 — Materials and methods used for supporting figures. (0.06 MB DOC) [file pbio.1000544.s008.doc]

**Supplemental Material and Methods**

*In situ hybridization*

Whole-mount in situ hybridization was performed as described in [3]. For *hgg1*, *ntl* and *dlx3* in situ hybridization, antisense RNA probes were synthesized from partial sequences of the respective cDNAs. Pictures were taken with a dissecting microscope (Olympus SZX 12) equipped with a QImaging Micropublisher 5.0 camera.

*Fluorescence recovery after photobleaching (FRAP)*

FRAP experiments were conducted at a Leica SP5 confocal microscope using a 63x/1.2 water immersion lens. Cells expressing palmitoylated GFP (GAP43-GFP *m*RNA, 50pg/embryo) were cultured for 20 min on Concanavalin A-coated cover-slips. Only tightly adhering and non-blebbing cells were used for the analysis as surface movements alters the characteristic recovery time and yields values for the diffusion coefficient influenced by directed motion. A portion of the cell membrane was bleached during 1.2 s using a 488 nm laser line at full laser-power. Recovery was monitored for 110 s, until a plateau was reached. Background intensity (I_B_) was subtracted from each image and the intensity was corrected for the loss of intensity (I_Σ_) due to bleaching during the recovery:

I=(I_bleachspot_-I_B_)/(I_Σ_-I_B_) Eq. S1

The intensity - time curve was then fitted to an exponential I(t) = (1-M)*(1-exp(-τ*t) to extract the half-time τ_0.5_ = ln(0.5)/τ of the fluorescence recovery [4]. τ_0.5_ was used to calculate the diffusion coefficient D according to D = (z*l)/(4 τ_0.5_) where l is the length of the bleached membrane segment and z is the bleaching depth of the lateral membrane [5]. z was determined using fixed cells by acquiring a y-stack after a segment has been bleached for 1.2 s at full laser-power (data not shown).

**Supplemental References**

1. Borghi N, Brochard-Wyart F (2007) Tether extrusion from red blood cells: integral proteins unbinding from cytoskeleton. Biophys J 93: 1369-1379.

2. Ferrer JM, Lee H, Chen J, Pelz B, Nakamura F, et al. (2008) Measuring molecular rupture forces between single actin filaments and actin-binding proteins. Proc Natl Acad Sci U S A 105: 9221-9226.

3. Montero JA, Carvalho L, Wilsch-Brauninger M, Kilian B, Mustafa C, et al. (2005) Shield formation at the onset of zebrafish gastrulation. Development 132: 1187-1198.

4. Liu LN, Aartsma TJ, Thomas JC, Zhou BC, Zhang YZ (2009) FRAP analysis on red alga reveals the fluorescence recovery is ascribed to intrinsic photoprocesses of phycobilisomes than large-scale diffusion. PLoS One 4: e5295.

5. Reits EA, Neefjes JJ (2001) From fixed to FRAP: measuring protein mobility and activity in living cells. Nat Cell Biol 3: E145-147.
